# Evaluation of Project P.A.T.H.S. (Secondary 2 Program) by the Program Participants: Findings Based on the Experimental Implementation Phase

**DOI:** 10.1100/tsw.2008.83

**Published:** 2008-05-23

**Authors:** Daniel T. L. Shek, Rachel C. F. Sun, Candace W. Y. Chan

**Affiliations:** ^1^ Department of Sociology, East China Normal University, Shanghai, Hong Kong; ^2^ Kiang Wu Nursing College of Macau, Macau; ^3^ Social Welfare Practice and Research Centre, The Chinese University of Hong Kong, Hong Kong

**Keywords:** adolescence, positive youth development, human development, Chinese, Hong Kong

## Abstract

A total of 49 schools participated in the Secondary 2 Program of the Experimental Implementation Phase of the Project P.A.T.H.S. (Positive Adolescent Training through Holistic Social Programmes). After completion of the program, 7,406 students completed a Subjective Outcome Evaluation Form (Form A) designed by the research team to reveal their comments about the program, instructors, and perceived effectiveness of the program. Based on the consolidated reports submitted by the schools, the research team aggregated the data to form a “reconstructed” overall profile on the perceptions of the program participants. Results showed that high proportions of the respondents had positive perceptions of the program and the instructors. About 80% of the respondents were satisfied with the program and regarded it as helpful to their overall development. The present findings provide support to the effectiveness of Secondary 2 Program of Project P.A.T.H.S. from the perspective of the program participants.

## INTRODUCTION

To promote holistic adolescent development in the young people of Hong Kong, the Hong Kong Jockey Club Charities Trust initiated and financially sponsored a multiyear, universal, positive youth development program entitled Project P.A.T.H.S. (**P**ositive **A**dolescent **T**raining through **H**olistic **S**ocial Programmes)[1,2]. For the Tier 1 Program, the research team developed a set of curriculum manuals that include materials based on 15 positive youth development constructs identified from the existing successful positive youth development programs[3,4], and took reference to relevant research findings and existing programs in both local and foreign contexts. The Tier 1 Program normally provides 20 h of training involving 40 teaching units for Secondary 1 to 3 students at each grade. In view of the schools' tight teaching timetable, the participating schools are also given the flexibility to implement the core program, which comprises 10 h of training involving 20 units.

There are two implementation phases in this project: the Experimental Implementation Phase and the Full Implementation Phase. In the 2005/06 school year, 52 secondary schools participated in the Secondary 1 Program of the Experimental Implementation Phase with the aim to accumulate experience in program implementation and to familiarize front-line workers with the program design and philosophy. In the 2006/07 school year, 49 out of these 52 schools continued to participate in the Secondary 2 Program of the Experimental Implementation Phase, whereas 207 schools joined the Secondary 1 Program of the Full Implementation Phase. Although there are papers documenting the positive program effects of the Tier 1 Program of the project[5,6,7,8,9,10,11,12,13,14,15,16,17], most of them are limited to the Secondary 1 Program. Only two papers were published to indicate the effectiveness of the Secondary 2 Program, and they were related to interim evaluation[18] and systematic observations of the delivery of the program[19]. However, these studies were process evaluation involving a small sample of schools and were based on the perspectives of program implementers and reports completed by observers. Based on the principles of triangulation, it is noteworthy to examine the views of program participants regarding the program and program outcome in order to give a full picture regarding program effectiveness.

In the review by Shek et al.[20], there were several developmental characteristics of Secondary 2 students. These included adjustment to puberty, cognitive maturation, rapid expansion of social circle, increased stress, encountering higher levels of social expectations, and gradual detachment from the family. Furthermore, compared with Secondary 1 students, Secondary 2 students had poorer psychological well-being[21,22], felt more helpless[23], had lower levels of life satisfaction[24], and they perceived family functioning to be poorer, parental control to be looser, and their relationships with parents to be worse[24,25,26,27,28,29]. Utilizing longitudinal research findings, Shek[30,31] showed that while parental behavioral control and parent-child relational qualities declined in the early adolescent years, parental psychological control increased.

With respect to the characteristics and psychosocial needs of Secondary 2 students, relatively more units of the Secondary 2 curriculum were designed to focus on cultivating the students' development in the individual and society domains with reference to the ecological perspective[32]. For example, the students were encouraged to live a meaningful life and to participate in healthy community activities. Moreover, 18 units were designed to tackle some current youth issues, such as mental health problems, substance abuse and smoking, heterosexual relationships, and materialism. In connection with this, it is pertinent to ask whether the reactions of Secondary 2 students to the Tier 1 Program would be different from those based on the Secondary 1 Program. Therefore, subjective outcome evaluation based on the views of the students should be conducted.

In the present study, the data collected from 49 schools participating in the Secondary 2 Program of the Experimental Implementation Phase were examined. As the Project P.A.T.H.S. was financially supported by The Hong Kong Jockey Club Charities Trust, each participating school had to submit an evaluation report with the consolidated subjective outcome evaluation profile of the school to the funding body. Such reports were then used by the research team to "reconstruct" the overall profile of the subjective outcome evaluation data. The major advantage of this strategy is to promote practice evaluation in the field and to conduct secondary data analysis of the reports submitted at the same time.

## METHODS

### Participants and Procedures

There were 49 schools that joined the Secondary 2 Program of the Experimental Implementation Phase. The mean number of students per school was 166.67 (range: 32–240 students), with an average of 4.51 classes per school (range: 1–7 classes). Among them, 22 schools adopted the full program (i.e., 20-h program involving 40 units) and the other 27 schools adopted the core program (i.e. 10-h program involving 20 units). The mean number of sessions used to implement the program was 23.76 (range: 10–

40 sessions). While 26 (53.06%) schools incorporated the program in the formal curriculum (e.g., Liberal Studies, Life Education), 23 schools (46.94%) used other modes (e.g., form master's periods and other combinations) to implement the program. The mean numbers of social workers and teachers implementing the program per school were 2.27 (range: 0–6) and 4.55 (range: 0–12), respectively.

After completion of the Tier 1 Program, a total of 7,406 students (mean = 151.14 students per school, range: 32–220) responded to the Subjective Outcome Evaluation Form (Form A) developed by the research team. On the day of data collection, which normally occurs at the last session of the program, the purpose of the evaluation was mentioned and the confidentiality of the data collected was repeatedly emphasized to all of the students. "Passive" informed consent was obtained from the students. Adequate time was provided for the participants to complete the questionnaire in a self-administration format and in an anonymous manner. To facilitate the program evaluation, the research team developed an evaluation manual with standardized instructions for collecting the subjective outcome evaluation data[33]. In addition, adequate training was provided to the workers during 20-h training workshops on how to collect and analyze the data collected by Form A[34].

## Instruments

The Subjective Outcome Evaluation Form (Form A) designed by Shek and Siu[33] consisted of several parts:

- Participants' perceptions of the program, such as program objectives, design, classroom atmosphere, interaction among the students, and the respondents' participation during class (10 items).
- Participants' perceptions of the workers, such as the preparation of the instructor, professional attitude, involvement, and interaction with the students (10 items).
- Participants' perceptions of the effectiveness of the program, such as promotion of different psychosocial competencies, resilience, and overall personal development (16 items).
- The extent to which the participants would recommend the program to other people with similar needs (1 item).
- The extent to which the participants would join similar programs in the future (1 item).
- Overall satisfaction with the program (1 item).
- Things that the participants learned from the program (open-ended question).
- Things that the participants appreciated most (open-ended question).
- Opinion about the instructor(s) (open-ended question).
- Areas that require improvement (open-ended question).

For the closed-end questions, the program workers were requested to input the collected data into an EXCEL file developed by the research team, which would automatically compute the frequencies and percentages associated with the different ratings for an item. The schools were also requested to submit the soft copy of the consolidated data sheets when they submitted the reports to the funding body. After receiving the consolidated data by the funding body, the research team aggregated the data to "reconstruct" the overall profile based on the subjective outcome evaluation data.

## RESULTS

Reliability analysis with the schools as the unit of analyses showed that Form A was internally consistent: 10 items related to the program (alpha = 0.98, mean interitem correlation = 0.84), 10 items related to the instructor (alpha = 0.99, mean interitem correlation = 0.94), 16 items related to the effectiveness (alpha =

0.99, mean interitem correlation = 0.93), and 39 items based on whole Form A (alpha = 0.99, mean interitem correlation = 0.82).

The quantitative findings based on the closed-ended questions are presented in this paper. There are several observations that can be highlighted from the findings. First, about 70% of the respondents perceived the program positively (Table 1), including the clear objectives of the curriculum (79.4%), systematic planning of activities (77.9%), peer interaction among students (77.5%), and active involvement of students during class (76.6%). Second, a high proportion of the students (84.9%) had positive evaluation of the instructors (Table 2). For example, about 86% of the respondents thought that the instructor was very involved; about 85% indicated that the instructor was well-prepared for the lessons, encouraged students to participate in the activities, and was ready to provide help to students in need. Third, about four-fifths of the respondents perceived that the program promoted their overall development (Table 3), including ability to resist harmful influences (79.4%), ability to distinguish between the good and the bad (81.4%), competence in making sensible and wise choices (79.7%), and compassion and care about others (79.3%). Fourth, as shown in Table 4, while 80% of the participants were satisfied with the course and more than 70% of the participants would recommend the program to their friends who have similar needs, only a simple majority of them (60%) would join similar programs in the future.

## DISCUSSION

This study aimed to evaluate the Secondary 2 Program of the Project P.A.T.H.S. via subjective outcome evaluation based on the perspective of the program participants. Generally speaking, the quantitative findings showed that a high proportion of the students had positive perceptions of the program and the workers, and about four-fifths of them regarded the program as beneficial to their development. Although the percentages of positive responses decreased in most items of the subjective outcome evaluation when compared with those reported by the participants when they were in Secondary 1[9] (e.g., the percentage of students indicated that they liked the curriculum dropped from 72.8 to 70.4%), the drops were trivial. On the whole, the present findings replicated previous subjective outcome evaluation findings of the Secondary 1 Program of the Experimental Implementation Phase[9,10,11,12] and those of the Full Implementation Phase[35,36]. Also, based on the principal of triangulation and utilization-focused evaluation[37], the present findings were in conjunction with the findings obtained from the interim evaluation[18] and systematic observations[19] of the Secondary 2 Program of the Experimental Implementation Phase, and showed that all the stakeholders had favorable perceptions of the program and high ratings of helpfulness of the program.

Although utilization of subjective outcome evaluation has been criticized as biased and unable to reflect the real behavioral changes in the program participants[38,39,40,41], there are several strengths of this study. First, this study used a big sample size (7,406 students in 49 participating schools) that substantially enhanced the generalizability and credibility of the research findings. Second, this study investigated different aspects of subjective outcome, including views on the program, instructors, perceived effectiveness, and overall satisfaction, and all these scales were found to be reliable. In fact, when the reliability of the different measures were compared with those reported elsewhere[35], the psychometric properties of Form A were highly comparable across studies. Third, as the findings reported in this paper were “reconstructed” based on the reports submitted by the participating schools anonymously, the possibility that the students reported in an overcooperative manner was not high. Finally, previous research findings based on the study have shown that subjective outcome evaluation findings actually converged with objective outcome evaluation findings[42,43].

Nevertheless, there are three alternative explanations for the present positive outcomes: (1) students were frightened of punishment if they voiced any unfavorable views, (2) students responded cooperatively in order to help the workers to illustrate positive program effect, and (3) students did not respond in a serious manner, resulting in a random and high proportion of positive responses. However,

**TABLE 1**
**Views of the Program Participants about the Program**

| | 1 | | 2 | | 3 | | 4 | | 5 | | 6 | | Participants with Positive Responses (Option 4–6) | |
|---|---|---|---|---|---|---|---|---|---|---|---|---|---|---|
| | Strongly Disagree | | Disagree | | Slightly Disagree | | Slightly Agree | | Agree | | Strongly Agree | | | |
| | N | % | N | % | N | % | N | % | N | % | N | % | N | % |
| The objectives of the curriculum are very clear. (N = 7,348) | 263 | 3.58 | 332 | 4.52 | 918 | 12.49 | 2,637 | 35.89 | 2,586 | 35.19 | 612 | 8.33 | 5,835 | 79.41 |
| The design of the curriculum is very good. (N = 7,338) | 279 | 3.80 | 450 | 6.13 | 1,148 | 15.64 | 2,854 | 38.89 | 2,120 | 28.89 | 487 | 6.64 | 5,461 | 74.42 |
| The activities were carefully planned. (N = 7,335) | 243 | 3.31 | 382 | 5.21 | 995 | 13.57 | 2,846 | 38.80 | 2,378 | 32.42 | 491 | 6.69 | 5,715 | 77.91 |
| The classroom atmosphere was very pleasant. (N = 7,304) | 317 | 4.34 | 435 | 5.96 | 1,086 | 14.87 | 2,518 | 34.47 | 2,162 | 29.60 | 786 | 10.76 | 5,466 | 74.84 |
| There was much peer interaction among the students. (N = 7,284) | 279 | 3.83 | 389 | 5.34 | 974 | 13.37 | 2,422 | 33.25 | 2,324 | 31.91 | 896 | 12.30 | 5,642 | 77.46 |
| I participated actively during lessons (including discussions, sharing, games, etc.). (N = 7,319) | 307 | 4.19 | 392 | 5.36 | 1,014 | 13.85 | 2,486 | 33.97 | 2,291 | 31.30 | 829 | 11.33 | 5,606 | 76.60 |
| I was encouraged to do my best. (N = 7,322) | 324 | 4.43 | 487 | 6.65 | 1,210 | 16.53 | 2,697 | 36.83 | 2,058 | 28.11 | 546 | 7.46 | 5,301 | 72.40 |
| The learning experience I encountered enhanced my interest towards the lessons. (N = 7,309) | 354 | 4.84 | 530 | 7.25 | 1,188 | 16.25 | 2,661 | 36.41 | 2,015 | 27.57 | 561 | 7.68 | 5,237 | 71.65 |
| Overall speaking, I have very positive evaluation of the program. (N = 7,329) | 393 | 5.36 | 518 | 7.07 | 1,220 | 16.65 | 2,632 | 35.91 | 2,024 | 27.62 | 542 | 7.40 | 5,198 | 70.92 |
| On the whole, I like this curriculum very much. (N = 7,296) | 482 | 6.61 | 545 | 7.47 | 1,134 | 15.54 | 2,424 | 33.22 | 2,040 | 27.96 | 671 | 9.20 | 5,135 | 70.38 |

**TABLE 2**
**Views of the Program Participants about the Program Implementers**

| | 1 Strongly Disagree | | 2 Disagree | | 3 Slightly Disagree | | 4 Slightly Agree | | 5 Agree | | 6 Strongly Agree | | Participants with Positive Responses (Option 4–6) | |
|---|---|---|---|---|---|---|---|---|---|---|---|---|---|---|
| | N | % | N | % | N | % | N | % | N | % | N | % | N | % |
| The instructor(s) had a good mastery of the curriculum. (N = 7,346) | 244 | 3.32 | 270 | 3.68 | 721 | 9.81 | 2,488 | 33.87 | 2,758 | 37.54 | 865 | 11.78 | 6,111 | 83.19 |
| The instructor(s) was well prepared for the lessons. (N = 7,342) | 194 | 2.64 | 237 | 3.23 | 638 | 8.69 | 2,276 | 31.00 | 2,902 | 39.53 | 1,095 | 14.91 | 6,273 | 85.44 |
| The instructor(s)' teaching skills were good. (N = 7,339) | 222 | 3.02 | 251 | 3.42 | 842 | 11.47 | 2,397 | 32.66 | 2,700 | 36.79 | 927 | 12.63 | 6,024 | 82.08 |
| The instructor(s) showed good professional attitudes. (N = 7,335) | 217 | 2.96 | 237 | 3.23 | 696 | 9.49 | 2,292 | 31.25 | 2,786 | 37.98 | 1,107 | 15.09 | 6,185 | 84.32 |
| The instructor(s) was very involved. (N = 7,320) | 211 | 2.88 | 208 | 2.84 | 600 | 8.20 | 2,207 | 30.15 | 2,818 | 38.50 | 1,276 | 17.43 | 6,301 | 86.08 |
| The instructor(s) encouraged students to participate in the activities. (N = 7,330) | 215 | 2.93 | 200 | 2.73 | 666 | 9.09 | 2,221 | 30.30 | 2,769 | 37.78 | 1,259 | 17.18 | 6,249 | 85.25 |
| The instructor(s) cared for the students. (N = 7,330) | 238 | 3.25 | 250 | 3.41 | 760 | 10.37 | 2,272 | 31.00 | 2,758 | 37.63 | 1,052 | 14.35 | 6,082 | 82.97 |
| The instructor(s) was ready to offer help to students when needed. (N = 7,315) | 207 | 2.83 | 229 | 3.13 | 650 | 8.89 | 2,219 | 30.33 | 2,848 | 38.93 | 1,162 | 15.89 | 6,229 | 85.15 |
| The instructor(s) had much interaction with the students. (N = 7,328) | 261 | 3.56 | 276 | 3.77 | 855 | 11.67 | 2,364 | 32.26 | 2,574 | 35.13 | 998 | 13.62 | 5,936 | 81.00 |
| Overall speaking, I have very positive evaluation of the instructors. (N = 7,346) | 270 | 3.68 | 223 | 3.04 | 619 | 8.43 | 2,113 | 28.76 | 2,872 | 39.10 | 1,249 | 17.00 | 6,234 | 84.86 |

all these alternative explanations could be dismissed because (1) student participation was voluntary and anonymous, (2) negative ratings were in fact recorded (e.g., whether the participants would join the program again), and (3) reliability analyses showed that the whole scale was internally consistent.

On the other hand, there are some limitations of the study. First, characteristics at the individual program participant level cannot be examined because the data were reconstructed from the reports submitted by the schools and, thus, the unit of analysis was schools rather than individual students. Second,

**TABLE 3**
**Perceived Effectiveness of the Program by the Program Participants**

| The extent to which the course has helped you. | 1 Unhelpful | | 2 Not Very Helpful | | 3 Slightly Helpful | | 4 Helpful | | 5 Very Helpful | | Participants with Positive Responses (Option 3–5) | |
|---|---|---|---|---|---|---|---|---|---|---|---|---|
| | N | % | N | % | N | % | N | % | N | % | N | % |
| It has strengthened my bonding with teachers, classmates and my family. (N = 7,331) | 512 | 6.98 | 1,416 | 19.32 | 3,154 | 43.02 | 1,809 | 24.68 | 440 | 6.00 | 5,403 | 73.70 |
| It has strengthened my resilience in adverse conditions. (N = 7,335) | 411 | 5.60 | 1,283 | 17.49 | 2,997 | 40.86 | 2,058 | 28.06 | 586 | 7.99 | 5,641 | 76.91 |
| It has enhanced my social competence. (N = 7,320) | 416 | 5.68 | 1,133 | 15.48 | 2,939 | 40.15 | 2,199 | 30.04 | 633 | 8.65 | 5,771 | 78.84 |
| It has improved my ability in handling and expressing my emotions. (N = 7,319) | 425 | 5.81 | 1,138 | 15.55 | 2,881 | 39.36 | 2,228 | 30.44 | 647 | 8.84 | 5,756 | 78.64 |
| It has enhanced my cognitive competence. (N = 7,314) | 425 | 5.81 | 1,149 | 15.71 | 2,866 | 39.19 | 2,209 | 30.20 | 665 | 9.09 | 5,740 | 78.48 |
| My ability to resist harmful influences has been improved. (N = 7,322) | 426 | 5.82 | 1,082 | 14.78 | 2,817 | 38.47 | 2,250 | 30.73 | 747 | 10.20 | 5,814 | 79.40 |
| It has strengthened my ability to distinguish between the good and the bad. (N = 7,314) | 374 | 5.11 | 987 | 13.49 | 2,869 | 39.23 | 2,323 | 31.76 | 761 | 10.40 | 5,953 | 81.39 |
| It has increased my competence in making sensible and wise choices. (N = 7,316) | 402 | 5.49 | 1,081 | 14.78 | 2,849 | 38.94 | 2,276 | 31.11 | 708 | 9.68 | 5,833 | 79.73 |
| It has helped me to have life reflections. (N = 7,319) | 494 | 6.75 | 1,069 | 14.61 | 2,749 | 37.56 | 2,146 | 29.32 | 861 | 11.76 | 5,756 | 78.64 |
| It has reinforced my self-confidence. (N = 7,310) | 506 | 6.92 | 1,260 | 17.24 | 2,726 | 37.29 | 2,080 | 28.45 | 738 | 10.10 | 5,544 | 75.84 |
| It has increased my self-awareness. (N = 7,317) | 483 | 6.60 | 1,149 | 15.70 | 2,797 | 38.23 | 2,182 | 29.82 | 706 | 9.65 | 5,685 | 77.70 |
| It has helped me to face the future with a positive attitude. (N = 7,312) | 469 | 6.41 | 1,141 | 15.60 | 2,734 | 37.39 | 2,214 | 30.28 | 754 | 10.31 | 5,702 | 77.98 |
| It has helped me to cultivate compassion and care about others. (N = 7,308) | 493 | 6.75 | 1,018 | 13.93 | 2,845 | 38.93 | 2,209 | 30.23 | 743 | 10.17 | 5,797 | 79.32 |
| It has encouraged me to care about the community. (N = 7,308) | 557 | 7.62 | 1,191 | 16.30 | 2,802 | 38.34 | 2,080 | 28.46 | 678 | 9.28 | 5,560 | 76.08 |
| It has promoted my sense of responsibility in serving the society. (N = 7,320) | 511 | 6.98 | 1,152 | 15.74 | 2,803 | 38.29 | 2,132 | 29.13 | 722 | 9.86 | 5,657 | 77.28 |
| It has enriched my overall development. (N = 7,314) | 466 | 6.37 | 961 | 13.14 | 2,679 | 36.63 | 2,318 | 31.69 | 890 | 12.17 | 5,887 | 80.49 |

while the reconstructed profile can give some ideas about the global picture, those unfavorable responses were diluted. Third, the restricted response format of the closed-ended questions would limit the respondents' expression of negative experiences, and the quantitative findings would hardly unravel the inner world of the respondents[44,45]. Hence, in order to illuminate the quantitative findings, further effort to examine such negative responses by looking at the qualitative findings is necessary. Fourth, as three out of the 52 schools that participated in Secondary 1 Program of the Experimental Implementation Phase refused to join the Secondary 2 Program, it is important to understand the relationship between school drop-out and the evaluation of program effectiveness.

**TABLE 4**
**Other Aspects of Subjective Outcome Evaluation based on the Views of the Program Participants**

If your friends have needs and conditions similar to yours, will you suggest him/her to join this course? (N = 7,257)

| 1 | | 2 | | 3 | | 4 | | Participants with Positive Responses (Option 3–4) | |
|---|---|---|---|---|---|---|---|---|---|
| Definitely Will Not Suggest | | Will Not Suggest | | Will Suggest | | Definitely Will Suggest | | | |
| N | % | N | % | N | % | N | % | N | % |
| 667 | 9.19 | 1269 | 17.49 | 4512 | 62.17 | 809 | 11.15 | 5321 | 73.32 |

Will you participate in similar courses again in the future? (N = 7,253)

| 1 | | 2 | | 3 | | 4 | | Participants with Positive Responses (Option 3–4) | |
|---|---|---|---|---|---|---|---|---|---|
| Definitely Will Not Participate | | Will Not Participate | | Will Participate | | Definitely Will Participate | | | |
| N | % | N | % | N | % | N | % | N | % |
| 964 | 13.29 | 1935 | 26.68 | 3616 | 49.86 | 738 | 10.18 | 4354 | 60.03 |

On the whole, are you satisfied with this course? (N = 7,250)

| 1 | | 2 | | 3 | | 4 | | 5 | | 6 | | Participants with Positive Responses (Option 4–6) | |
|---|---|---|---|---|---|---|---|---|---|---|---|---|---|
| Very Dissatisfied | | Moderately Dissatisfied | | Slightly Dissatisfied | | Satisfied | | Moderately Satisfied | | Very Satisfied | | | |
| N | % | N | % | N | % | N | % | N | % | N | % | N | % |
| 393 | 5.42 | 355 | 4.90 | 695 | 9.59 | 3,431 | 47.32 | 1,724 | 23.78 | 652 | 8.99 | 5,807 | 80.10 |

Despite these limitations, the present findings fill up the research gap of the perceived effectiveness of the Secondary 2 Program, and suggest that the Tier 1 Program and its implementation were perceived in a positive manner by the program participants. With the gradual decline of parental influence and adolescent psychological well-being in the early adolescent years, the perceived benefits of the program suggest that the use of the Tier 1 Program can help Secondary 2 students to develop psychosocial competencies and enhance their holistic development in the Chinese context.

## ACKNOWLEDGMENTS

The preparation for this paper and the Project P.A.T.H.S. were financially supported by the Hong Kong Jockey Club Charities Trust.
